# Association of Infrastructure and Route Environment Factors with Cycling Injury Risk at Intersection and Non-Intersection Locations: A Case-Crossover Study of Britain

**DOI:** 10.3390/ijerph18063060

**Published:** 2021-03-16

**Authors:** Rachel Aldred, Georgios Kapousizis, Anna Goodman

**Affiliations:** 1 School of Architecture and Cities, Westminster University, London NW1 5LS, UK; 2 Faculty of Engineering Technology, University of Twente, 7500 AE Enschede, The Netherlands; g.kapousizis@utwente.nl; 3 London School of Hygiene & Tropical Medicine, Faculty of Epidemiology and Population Health, London WC1E 7HT, UK; anna.goodman@lshtm.ac.uk

**Keywords:** cycling, injury, route environment, case-crossover, infrastructure, intersections

## Abstract

Objective: This paper examines infrastructural and route environment correlates of cycling injury risk in Britain for commuters riding in the morning peak. Methods: The study uses a case-crossover design which controls for exposure. Control sites from modelled cyclist routes (matched on intersection status) were compared with sites where cyclists were injured. Conditional logistic regression for matched case–control groups was used to compare characteristics of control and injury sites. Results: High streets (defined by clustering of retail premises) raised injury odds by 32%. Main (Class A or primary) roads were riskier than other road types, with injury odds twice that for residential roads. Wider roads, and those with lower gradients increased injury odds. Guard railing raised injury odds by 18%, and petrol stations or car parks by 43%. Bus lanes raised injury odds by 84%. As in other studies, there was a ‘safety in numbers’ effect from more cyclists. Contrary to other analysis, including two recent studies in London, we did not find a protective effect from cycle infrastructure and the presence of painted cycle lanes raised injury odds by 54%. At intersections, both standard and mini roundabouts were associated with injury odds several times higher than other intersections. Presence of traffic signals, with or without an Advanced Stop Line (‘bike box’), had no impact on injury odds. For a cyclist on a main road, intersections with minor roads were riskier than intersections with other main roads. Conclusions: Typical cycling environments in Britain put cyclists at risk, and infrastructure must be improved, particularly on busy main roads, high streets, and bus routes.

## 1. Introduction

While there is much work looking at cycling injuries, relatively little considers injury risk as opposed to injury numbers or injury severity (e.g., [1]). More research is needed that incorporates exposure or amount of cycling, so that we can separate the risk that a (type of) location poses to each cyclist from the number of cyclists using that (type of) location.

For example, one might observe a comparatively large number of injuries on a popular cycling route, but without knowing how many cyclists use that route (exposure) it would be unclear whether the risk per cyclist was higher, lower or equivalent to surrounding streets. This could lead to incorrect decisions about infrastructure interventions: for instance, planners might believe that an infrastructure intervention was dangerous based on absolute injury numbers, when in fact it might reduce injuries per cyclist (or vice versa).

Part of the reason for the lack of risk-based analysis is a paucity of cycling flow data on which to base such exposure calculations [2]; this is compounded by a lack of good spatial data on route characteristics. Analysis controlling for exposure hence often focuses on a small selection of sites [3] to facilitate bespoke data collection. There are relatively few comprehensive analyses covering a range of sites and a range of infrastructure types, with some [4] using area-level data, limiting the ability to link risk directly to route segment characteristics.

There are some studies that control for exposure. These suggest that intersections and major roads are associated with higher injury risk (e.g., [5,6,7]), as is volume of motor traffic [7]. Lower speed limits may reduce risk [8] while hills and especially downhill gradients increase it [9]. Cycling infrastructure has been found to reduce risk per cyclist [6]. However, studies suggest that this may only be true for tracks protected/separated from motor traffic [10]. In London, protected cycle infrastructure was associated with a reduction in injury risk of 40–65%; however, painted advisory cycle lanes were associated with an increase of 30% [11].

More evidence is still needed, especially covering whole networks. Where aggregate exposure data exists, case–control methods can be used (e.g., [6,7,12]) however, at a national level such data is rarely available. This study uses a case-crossover design, allowing us to control for individual-level variation, unlike aggregate methods. Our first set of findings (author reference deleted) compared injury sites to randomly selected control sites. One finding was the high risk associated with intersection status (i.e., if a site was at or close to an intersection). Like [13] this paper presents a new set of results matched by intersection status, allowing investigation of risks associated with specific characteristics of intersection and non-intersection sites.

## 2. Materials and Methods

### 2.1. Approach

This paper examines correlates of cycling injury risk in Great Britain in 2017. Ethical approval was given by the University of Westminster.

As in [9] the study uses a case-crossover method. Researchers randomly generate control points from the routes followed by individual cyclists prior to experiencing an injury. This produces a set of control sites representing the typical types of route environment experienced by the injured cyclists; and the types of places they might instead have been injured were all types of route environment equally risky. Hence, comparing this set of matched controls with their cases (corresponding injury locations) allows researchers to establish which out of a range of characteristics of injury sites (e.g., road width, street infrastructure) are associated with increased odds of injury.

As we did not have actual cyclist routes (unlike [9]) we used the Cyclestreets fastest-route journey planner to model cyclist routes prior to injury. Comparison with observed cyclist routes and evidence from other published work (e.g., [14]) suggests that this predicts sufficiently well the types of routes that cyclists follow for trips such as commuting (directness being the major factor, but not the only one). (More information about the Cyclestreets journey planner can be found online: https://www.cyclestreets.net/help/journey/routing (accessed on 15 March 2021). We also do not know exactly where a cyclist might have been riding at any point: whether on the footway, using or not using a cycle lane or bus lane. Thus, the study can tell us about the safety impact of the presence of certain types of infrastructure, not their actual use.

### 2.2. Data Sources

We obtained home postcode data from Department for Transport, for all cyclists injured in Britain during 2017. While we did also have data from Northern Ireland, this represented only ~1% of all cycle injuries, and much route environment data only covered Britain. Hence, we decided to only cover Britain in this analysis). This was necessary to generate routes and hence control locations. For many trips the start location is a person’s home, and this can be predicted based on trip timing given that that >95% of cycle trips during the morning peak start from home. We used home postcode data alongside publicly available Stats19 injury data, which includes the point co-ordinates of the collision location.

### 2.3. Generation of Routes and Set of Controls

In Britain between 5 a.m. and 9:59 a.m., Monday to Friday, 4303 cyclists were injured during 2017. Of these 3507 (81.5%) had full home postcode data. We used the Cyclestreets API (fastest-route option) to model routes from home postcode area centroids to the points of injury (set of cases). The use of postcode centroids rather than exact addresses will make little difference to results. Postcodes contain around 15 addresses, so in major urban conurbations with terraced or flatted housing (>90% of cases) this implies a very small segment of street. Even in smaller towns, each postcode contains a small cluster of nearby houses, generally all on the same street. The only possible exceptions would be sparse areas, but Census 2011 data shows that under 1% of cycle commuters live in such areas.

We excluded points associated with routes longer than 25 km (137 routes, or 3.9%) as we considered these unlikely to have started at the person’s home location. We excluded 29 points where injury occurred <100 m from home, as this did not give sufficient scope for the control and injury point to differ in their characteristics.

We initially generated one control point location (set of controls) randomly from each of the 3341 remaining routes, using ArcGIS Random Points. As explained in more detail, when randomly generating controls, we ensured that all fell on or adjacent to the highway network, because the Stats19 injury dataset from which the cases are obtained only contains injuries sustained in such locations.

Initial analysis showed strong associations between intersection status and injury risk. Specifically, when comparing our set of combined cases and controls (*n* = 6682, 50% cases and 50% controls) we found that cases made up 62% of the intersection sites, but only 29% of the non-intersection sites. This was a univariate odds ratio of 4.42 (95% CI 3.90, 5.00), or 3.43 (2.99, 3.93) after adjusting for area, road, street infrastructure and vehicle variables. In analyses restricted only to KSI injuries (fatal and serious), the effect was 3.77 (2.68, 5.29).

These strong effects matched our expectation that intersection status would be a major predictor of odds of injury, supporting our decision to generate a set of control points matched on intersection status for these analyses. Some factors not associated with risk away from intersections might prove more problematic close to junctions, and vice versa, given different conflict profiles, for instance related to vehicle movements.

### 2.4. Route Environment Data

Our analysis is based on analysis of 3341 injury and 3341 control points. We sourced route environment data in a range of ways. This included datasets provided by partners (e.g., Basemap: Guildford, UK) or available online (e.g., OpenStreetMap, data generated globally from citizen mapping) and use of Google Street View. For details see Supplementary Table S1.

We assigned each point the following route environment characteristics, grouped a priori into four different categories:

1. Area type: urban/rural status, high street status (defined by clustering of retail premises), average small area deprivation.
2. Road type: road class, road width, road gradient, speed limit, street connectivity for motor vehicles within the network.
3. Nearby street infrastructure: Bicycle infrastructure, guard railing, bus lane, bus stop, metro/rail/tram stop, petrol station/car park, intersection status (proximity).
4. Vehicle factors: average AM peak speed, parked cars, cycle commuter flow.

Supplementary Table S1 presents details of how each variable was calculated. Note that while variables related to factors such as weather condition and road surface condition are present in Stats19 data, such factors cannot be included in our regression analysis as we do not have corresponding data from the set of control sites. The same applies to demographic and involved-vehicle factors, which are used to provide context, but which cannot be used in comparison between case and control sites.

### 2.5. Statistical Modelling

We used conditional logistic regression, matching each injury point to its sampled control point matched on intersection status. We analysed our data guided by our four-category classification of environmental correlates into area type, road type, nearby street infrastructure, and vehicle factors.

We fitted the adjusted regression models using a hierarchal modelling structure, starting with the categories of variables conceptualised as most distal to the outcome, and continuing with categories of variables we saw as mediating more distal factors. In stratified analyses restricted to intersection points, we included variables on traffic signals and roundabouts, as additional elements of street infrastructure. We included road type and vehicle factors variables for the intersecting road where available. We conducted sensitivity analyses restricted to KSI casualties, and present results for tests for interaction between each predictor and whether the injury was a KSI or not.

As our study is focusing on injuries occurring during the morning commute, control points will be closer to home and further from work than injury points, and on average places where people work are less residential and more commercial. Hence, we expected that injury locations would generally have a higher workplace density than control locations, as an artefact. This was indeed observed: workplace density was higher in the injury point for 1155 participants (34.6%), in the control point for 735 participants (22.0%), and similar (within 0.05) for 1451 participants (43.4%). To reduce confounding, we included workplace density in all adjusted models as a covariate.

Note also that the Propensity to Cycle Tool (PCT) route network (used to look up cycling volume, see Supplementary Table S1) was created using an algorithm (Cyclestreets) to route cyclists between origin and destination, based largely but not only on directness. By contrast injuries can happen anywhere that cyclists travel. Our method therefore means control points are less likely to be ‘off the PCT network’, and therefore less likely to get a zero or very low cycle volume value. For this reason, when modelling cycle volume as a continuous variable we simultaneously entered a binary dummy variable identifying whether the route contained 0–5 versus 6+ cyclists.

We examined crude associations to guide how continuous variables should be entered into our model. Motor connectivity ranking was highly correlated with road class and other road type variables, so we entered it as a categorical variable. Otherwise, where possible we entered continuous variables as linear terms, to increase power and avoid complications of interpretation from using quadratic terms. To limit the effect of outliers, we capped road width at 15 m (276 higher values, or 4.1%, rounded to 15), average peak speed at 50 miles/h (303 higher values, or 4.5%, rounded to 50) and the number of cycle commuters at 1000 (88 higher values, or 1.3%, rounded to 1000). After this, all continuous variables showed an approximately linear relationship in visual inspection, with no evidence of non-nonlinearity as judged by the inclusion of a quadratic term (all *p* > 0.05 in adjusted analyses).

The proportion of variables with missing data ranged from 0 to 6.2% with respect to the road on which the crash happened. At intersections, the proportion with missing data ranged from 0 to 12.6% with respect to the second, intersecting road. We imputed this data using multiple imputation (25 imputations) under an assumption of Missing at Random. We confirmed in sensitivity analyses that results were similar when using a complete case analysis on the 2589 participants (77.5%) with complete data for both injury and control points.

## 3. Results

### 3.1. Sample Characteristics

Characteristics of the 3341 individuals in our sample are shown in Table 1. The large majority were from England. 77% were male, 73% aged 25–59, and people living in the richest two-fifths of areas were somewhat underrepresented. 82% of injuries were slight, 17% serious and 0.4% fatal. The large majority, 91%, involved cars, taxis, or vans; 4% were ‘no other vehicle’ collisions. Most occurred when it was light (as expected given during the morning commute), in fine weather with dry road conditions.

### 3.2. Effects of Area, Road, Street Infrastructure and Vehicle Factors

Table 2 provides results from our modelling of injury predictors for all points (matched for intersection status).

#### 3.2.1. Effects of Area-Type Variables

Being urban and on a high street were both significantly associated with increased odds of injury in univariable analyses, but there was no association with area deprivation. The impact of being in an urban area attenuated and became no longer significant after mutual adjustment for presence of a high street (adjusted model 1), and then further attenuated upon additional adjustment. This suggests the univariable urban effect reflected the types of roads found in urban areas plus the higher concentration of high streets. The impact of being on a high street was somewhat attenuated after adjusting for road type, street infrastructure and vehicle factors, but a significant independent effect remained in the final adjusted model (3), suggesting some risk posed by aspects of the high street not captured in other variables (odds ratio 1.32, or a 32% increase in the odds of injury).

#### 3.2.2. Effects of Road Type Variables

All five variables were significantly associated with the odds of injury in univariable analyses. After mutual adjustment plus adjusting for area type and nearby street infrastructure (adjusted model 2), injury was independently predicted by primary road type; greater road width; and a lower gradient value (i.e., higher odds of injury for downhill travel than flat travel, and for flat travel than uphill travel). There was no longer evidence in adjusted analysis of an independent effect of speed limit or motor connectivity.

#### 3.2.3. Effects of Street Infrastructure Variables

Five of six variables were significantly associated with odds of injury in univariate analyses, the exception being a nearby bus stop. After mutual adjustment, plus adjusting for area type and road type (adjusted model 2), injury was independently predicted by the presence of a cycle lane (=on-road) or a cycle track (=off-road) plus a lane, but not a track alone. Note that ‘track’ here is used to describe any kind of highway-adjacent off-road infrastructure: in the UK, such infrastructure traditionally would tend to involve a shared footway rather than what would usually be known as a ‘cycle track’ on the Dutch or Danish model. Note also that we do not know whether a cyclist was in fact riding on provided infrastructure; thus, the findings cannot show whether use of typical UK cycle infrastructure is protective or risky, but rather whether its presence is.

These associations with cycle infrastructure type changed little after adjusting for vehicle factors variables (motor traffic speeds, parked cars, commuter cyclist flow). Increased injury odds was independently predicted by presence of a bus lane, guardrail, petrol station or car park. Again, none of these associations changed much after adjusting for the vehicle factors variables.

#### 3.2.4. Effects of Vehicle Factors Variables

Higher average traffic speed was associated with lower odds of injury in both univariate and adjusted analyses. Parked cars were associated with lower odds of injury in univariable analyses, but this effect disappeared in adjusted models, in particular after adjusting for road type (parked cars are more common on residential streets). In univariable analyses there was no association between odds of injury and volume of cycling, but after adjustment for other factors a higher volume of cyclists was associated with lower odds of injury.

### 3.3. Examination of Differential Effects between Slight Injuries versus KSI

We conducted stratified analyses comparing the 2749 individuals with a slight injury to the 592 individuals who were killed or seriously injured (KSI) (see Supplementary Materials Tables S2–S4). In general, the point estimates were similar between the two injury types, although less often statistically significant for KSI because of the much smaller sample size. There was never evidence of an interaction between any of the 17 predictor variables shown in Table 2 and KSI status (all *p* ≥ 0.09).

### 3.4. Examination of Differential Effects between Non-Intersection versus Intersection Points

We conducted stratified analyses comparing the 684 individuals who were not injured at an intersection to the 2657 individuals who were injured at an intersection. The adjusted results are shown in Table 3. Note that at intersections, ‘first road’ refers to the road on which the cyclist was travelling at that point (based on our modelling of their route to that point), and the ‘second road’ is the joining road.

As the number of non-intersection injuries was relatively small, the confidence intervals are fairly wide and there is low power for testing for interactions. In 15 of the 17 variables tested, there was little or no evidence of an interaction in adjusted analyses (all *p* ≥ 0.09). There was, however, strong evidence of an interaction between intersection status and road class (*p* < 0.001) such that the protective effect of being on a secondary road and in particular tertiary road was stronger at intersection than at non-intersection points. There was strong evidence of an interaction between intersection status and average speed (*p* = 0.004), such that the somewhat increased risk associated with very low speeds (perhaps representative of congestion) was more pronounced at intersections than non-intersections.

In Table 3, adjusted model 2 for intersection points includes some variables applying to the second road. After adjusting for the characteristics of the first road, there was a significantly increased odds of injury if the second road was a minor (i.e., not primary) road, and a significantly increased odds of injury if the second road was wider. Further exploratory analyses indicated an interaction between the road class of the first road and the second road (*p* = 0.003), such that injury odds were increased if first road were a primary road and the second road a minor road specifically (adjusted OR 2.41, 95% CI 1.88 to 3.08, compared to first road and second road both primary: see Supplementary Materials Tables S2–S4).

There was no effect of having a traffic signal present at an intersection, with or without an ASL. There was, however, substantially higher odds of injury if the intersection involved a roundabout or a mini roundabout, with similar effects of these two sorts of roundabouts. Finally, there was evidence that the odds of injury at intersections increased as average speed on the second road increased.

## 4. Discussion

### 4.1. Summary Findings

High street status was associated with an elevated injury risk in final adjusted models, while urban area status was not, an initial effect becoming attenuated when adjusting for other variables. In adjusted models, injury risk was independently predicted by road type being primary, and by a more downhill gradient. Lower speed limits and lower motor traffic connectivity were initially associated with lower injury risk, but these effects were no longer statistically significant when adjusting for other variables. Increased road width was associated with increased injury risk in all models.

Findings suggest that injury risk is increased by width and classification of road, and by factors generating potentially conflicting movements by other road users—i.e., intersections, shops, petrol stations and car parks, and parked cars, although the presence of other cyclists reduced risk. Bus lanes, a principal form of provision for cycling on busy roads, increase injury risk, although this increased risk is somewhat mitigated close to bus stops. Perhaps surprisingly, on-road cycle lanes are associated with an increase in risk similar to presence of a bus lane combined with a bus stop, and off-road infrastructure did not appear to be protective. This contrasts with a similar study in London which found that cycle tracks separated from motor traffic and from pedestrians were protective; although in the London study advisory on-road lanes also increased risk [11].

When separating intersection and non-intersection points, type of intersection mattered: both roundabouts and mini roundabouts raised injury odds threefold at intersection locations. Signals, with or without on-road infrastructure of Advanced Stop Lines (‘bike boxes’) were not associated with increase or decrease in injury risk. At intersections, the negative impact of main roads and of low morning traffic speeds were heightened.

### 4.2. Limitations

We were only able to include weekday morning peak journeys, which may affect some of our results—for instance, the speed variable may mainly be applicable to an urban peak hour context with a limited speed profile. We had to exclude injured cyclists for whom home postcode was not known. Our data predominantly relates to slight injuries involving motor vehicles, these being most injuries recorded by the police.

Our use of a modelling algorithm to route the cyclists could lead to bias, for instance, if cyclists in practice make more use of residential roads than is suggested by the algorithm. However, use of a relatively direct route (the Cyclestreets ‘fast route’ algorithm prioritises directness, considering the delaying impacts of hills and traffic signals) is, we believe, likely to represent well enough cyclist routes, especially at commuting times. This is discussed in more detail in a related paper from this project (in press; Kapousizis, Goodman, and Aldred). More research using routes reported by injured cyclists, such as [9] would be helpful, particularly if it incorporated testing against algorithmically generated routes, although such research is expensive and logistically challenging.

We were limited in route environment data sources available, and use of current Google Streetview images may introduce bias, if for instance infrastructure has been built post-2017 (which might be more likely in previously more dangerous environments). Presence of parked cars is an imperfect proxy since the Google Streetview cars mainly travel off-peak. We did not have data on motor traffic volume, as this is only available for major roads (as in many countries), not across the whole network. The connectivity dataset used was likely to represent a poor proxy.

### 4.3. Strengths

We used national data and controlled for cyclist volume and individual characteristics, through the case-crossover approach used. This is unusual and represents an innovative use of secondary data, allowing the research to be conducted without potentially intrusive, costly, and time-consuming primary data collection.

### 4.4. Meanings of Our Findings and Policy Implications

Unsurprisingly, our findings confirmed that main roads and wider roads (likely to have more traffic lanes) are riskier for people cycling. Adjusting for these factors meant that the impact of speed limits became statistically insignificant, although in univariate modelling 30 mph limit roads were riskier than 20 mph limit roads. Our modelling of actual motor traffic speeds in the morning peak suggested that congestion might increase injury risk, with roads with very low motor traffic speeds seeing higher risks; although at intersections, second roads with very low speeds conversely decreased risk. (Note also that most of our injuries are slight, hence a study of more serious injuries might find different patterns related to the association between speed and injury severity). The finding for guard railing suggests that this (anti)pedestrian infrastructure may help to create a perception among drivers that they will not encounter conflict with non-motorized users [15].

The negative impact of environments with conflicting motor traffic movements appears clear in most cases; away from intersections this is likely to particularly relate to curbside activity. Restrictions on car parking and hence better visibility for people cycling might then account for the somewhat protective effect of bus stops (without a bus lane, which has a larger negative impact). As in other studies, we found a safety in numbers impact from other cyclists being present on the road segment; there did not appear to be a negative impact from conflicting movements in relation to other cyclists.

Our findings in relation to cycle infrastructure are contrary to other literature, which generally finds a protective impact (cf. a recent systematic review [16]; although note this excluded case–control and case-crossover studies). Assuming that our algorithm has not introduced bias (i.e., if cyclists are in reality more likely to use roads with cycle infrastructure than predicted by the Cyclestreets direct routing), we believe the explanation likely lies in the quality of the cycle infrastructure typically existing across Britain in 2017. England’s new Cycle Infrastructure Design Guidance (LTN 1/20) suggests that infrastructure quality may start to improve. In London, where a similar update to guidance was published six years ago and where better data on cycle infrastructure type is available, studies already show a reduction in risk from types of higher-quality separated infrastructure [10,11].

## 5. Conclusions

Improvements to infrastructure and road conditions are most needed in contexts with higher existing risks. If roundabouts are to remain, higher-quality designs are needed, drawing on research from contexts such as the Netherlands where roundabouts are safer for cyclists than in the UK (e.g., [17]). Main roads, high streets, and roads with bus lanes are all risky for cyclists, yet often serve key desire lines and destinations. Such routes should be prioritised for higher-quality cycling infrastructure, ensuring high-quality design at intersections where current infrastructure is currently most problematic. As cyclists are also at high risk on main roads when passing side road junctions, these designs should not just focus on protecting cyclists at primary–primary junctions, but also reducing risk at side roads (for instance, reducing the number and speeds of turning movements into and out of side roads). Making quieter streets more attractive and pleasant for cycling, for instance through low traffic neighbourhood-type schemes restricting through motor traffic, can also help to provide safe alternative cycle routes.

## Figures and Tables

**Table 1:** Characteristics of individuals and of their crash.

Characteristic	Level	*N* (%)
Full sample		3341 (100%)
Country	England	3159 (94.6%)
	Scotland	131 (3.9%)
	Wales	51 (1.5%)
Sex	Male	2579 (77.2%)
	Female	762 (22.8%)
Age	0–15	293 (8.9%)
	16–24	415 (12.5%)
	25–39	1276 (38.5%)
	40–59	1139 (34.4%)
	60–74	155 (4.7%)
	75+	34 (1.0%)
Small-area	Fifth 1 (richest)	546 (17.3%)
deprivation	Fifth 2	569 (18.0%)
of home	Fifth 3	642 (20.3%)
	Fifth 4	778 (24.6%)
	Fifth 5 (poorest)	623 (19.7%)
Injury severity	Fatal	14 (0.4%)
	Serious	578 (17.3%)
	Slight	2749 (82.3%)
Striking vehicle	No other vehicle	188 (5.6%)
	Cyclist	20 (0.6%)
	HGV	70 (2.1%)
	Bus	38 (1.1%)
	Other motor vehicle, mostly cars	3025 (90.5%)
Light conditions	Light	2933 (87.8%)
	Dark	408 (12.2%)
Weather	Fine, no high winds	2708 (85.4%)
conditions	Other	464 (14.6%)
Road surface	Dry	2401 (74.3%)
conditions	Other	832 (25.7%)

Numbers add to less than 3341 for some variables due to missing data: in these cases, the % is calculated relative to those with non-missing data.

**Table 2:** Predictors of injury, all points.

Category	Predictor	Level	*n* Points	% Injury Points	Univariable	Adjusted 1	Adjusted 2	Adjusted 3
**Area** **Type**	**Urban**	**Rural**	464	47%	1 *	1	1	1
		**Urban**	6218	50%	1.41 (1.01, 1.96)	1.31 (0.94, 1.83)	1.15 (0.80, 1.66)	1.19 (0.82, 1.74)
	**High Street**	**No**	5953	49%	1 ***	1 ***	1 ***	1 **
		**Yes**	729	61%	1.85 (1.55, 2.20)	1.58 (1.32, 1.89)	1.48 (1.22, 1.80)	1.32 (1.08, 1.62)
	**Average deprivation**	**Change per standard deviation increase**	-	-	1.03 (0.96, 1.11)	1.04 (0.97, 1.12)	1.02 (0.94, 1.10)	1.01 (0.93, 1.09)
**Road** **type**	**Road class**	**Primary**	2561	58%	1 ***		1 ***	1 ***
		**Secondary**	745	49%	0.54 (0.44, 0.66)		0.67 (0.53, 0.84)	0.68 (0.54, 0.86)
		**Tertiary**	1215	45%	0.43 (0.36, 0.51)		0.55 (0.45, 0.67)	0.55 (0.45, 0.67)
		**Residential or other**	2160	44%	0.44 (0.38, 0.51)		0.60 (0.49, 0.74)	0.50 (0.40, 0.63)
	**Road width**	**Change per 1 m increase**	-	-	1.16 (1.14, 1.19) ***		1.11 (1.08, 1.14) ***	1.10 (1.07, 1.13) ***
	**Gradient**	**Change per 1% increase in incline (downhill = negative)**	-	-	0.97 (0.94, 0.99) *		0.96 (0.94, 0.99) **	0.96 (0.93, 0.98) **
	**Speed limit**	**20 mph or less**	1244	47%	1 **		1	1
		**30 mph**	4633	51%	1.34 (1.12, 1.61)		0.95 (0.77, 1.18)	0.95 (0.77, 1.18)
		**40 mph**	**395**	52%	1.51 (1.13, 2.03)		0.90 (0.64, 1.26)	1.10 (0.77, 1.57)
		**over 40 mph**	347	50%	1.31 (0.95, 1.82)		0.91 (0.62, 1.32)	1.10 (0.74, 1.62)
	**Connectivity** **rank**	**0–24%**	310	42%	1 ***		1	1
		**25–49%**	622	43%	1.06 (0.80, 1.40)		1.04 (0.77, 1.40)	1.09 (0.80, 1.47)
		**50–74%**	1246	47%	1.31 (1.01, 1.70)		1.17 (0.89, 1.55)	1.33 (1.00, 1.76)
		**75–100%**	4217	53%	1.72 (1.34, 2.20)		0.96 (0.72, 1.28)	1.17 (0.87, 1.58)
**Nearby ** **Street ** **infrastructure**	**Bicycle** **infrastructure**	**None**	5203	48%	1 ***		1 ***	1 ***
		**Track (no lane)**	571	53%	1.29 (1.07, 1.56)		1.19 (0.97, 1.46)	1.18 (0.96, 1.45)
		**Lane (no track)**	626	60%	1.86 (1.53, 2.26)		1.48 (1.20, 1.84)	1.54 (1.24, 1.91)
		**Track and Lane**	84	69%	2.79 (1.70, 4.56)		2.46 (1.45, 4.16)	2.46 (1.44, 4.22)
		**Other, e.g., sign**	142	50%	1.13 (0.80, 1.59)		1.23 (0.85, 1.78)	1.39 (0.95, 2.03)
	**Guardrail**	**No**	5598	49%	1 ***		1 **	1 *
		**Yes**	1028	58%	1.54 (1.33, 1.78)		1.25 (1.07, 1.46)	1.18 (1.01, 1.39)
	**Bus lane**	**No**	6267	49%	1 ***		1 ***	1 ***
		**Yes**	359	68%	2.51 (1.95, 3.23)		1.81 (1.37, 2.39)	1.84 (1.39, 2.44)
	**Bus stop**	**No**	6016	50%	1		1 **	1 **
		**Yes**	666	47%	0.89 (0.76, 1.05)		0.75 (0.63, 0.90)	0.77 (0.64, 0.92)
	**Metro/rail/** **tram stop**	**No**	6642	50%	1 *		1	1
		**Yes**	40	70%	2.60 (1.25, 5.39)		1.72 (0.79, 3.76)	1.52 (0.68, 3.36)
	**Petrol station or** **car park**	**No**	6259	49%	1 ***		1 **	1 **
		**Yes**	423	58%	1.47 (1.19, 1.81)		1.48 (1.18, 1.85)	1.43 (1.14, 1.79)
**Vehicle factors**	**2-way average morning peak speed**	**Change per 10 mph increase**	-	-	0.81 (0.77, 0.86) ***			0.78 (0.73, 0.84) ***
	**Parked cars**	**No**	2649	52%	1 **			1
		**Yes**	3977	49%	0.86 (0.78, 0.96)			1.00 (0.88, 1.14)
	**No. cycle commuters on segment**	**Change per 100 cyclists increase**	-	-	0.99 (0.95, 1.03)			0.94 (0.90, 0.99) *

* *p* < 0.05, ** *p* < 0.01, *** *p* < 0.001 in tests for heterogeneity. Numbers in the N’ column add to less than 6682 points for some variables due to missing data. In all other columns all 6682 points are used, using multiple imputation. All adjusted models additionally adjust for workplace density, as linear and quadratic terms, and when examining number of commuters on the segment we additionally included a dummy variable ‘0–5 cycle commuters versus 6+’. Control point selected after matching for intersection status: see Supplementary Materials Tables S2–S4 for equivalent analyses using control point selected without regard for intersection status.

**Table 3:** Results separating intersection and non-intersection sites, and additional results for intersection points.

Category	Predictor	Level	Non-Intersection Points (*n* = 1366 Points)			Intersection Points (*n* = 5312 Points)				*P*-Value for Interaction with Intersection Status, Adjusted 1 Models
			***n* points**	**% Injury Points**	**Adjusted**	***n* points**	**% Injury Points**	**Adjusted 1**	**Adjusted 2**	
**Area ** **type**	**Urban**	**Rural**	177	48%	1	287	47%	1	1	*p =* 0.57
		**Urban**	1191	50%	1.91 (0.91, 3.99)	5027	50%	1.04 (0.66, 1.64)	1.11 (0.68, 1.80)	
	**High Street**	**No**	1284	49%	1 *	4669	49%	1 *	1 **	*p =* 0.43
		**Yes**	84	68%	1.79 (1.01, 3.19)	645	60%	1.28 (1.03, 1.59)	1.44 (1.15, 1.80)	
	**Average deprivation**	**Change per standard deviation**	-	-	0.89 (0.74, 1.07)	-	-	1.03 (0.95, 1.13)	1.04 (0.95, 1.14)	*p =* 0.18
**Road ** **type, ** **first ** **road**	**Road class**	**Primary**	434	56%	1 *	2127	58%	1 ** *	1 ***	*p <* 0.001
		**Secondary**	185	51%	0.85 (0.51, 1.40)	560	48%	0.63 (0.49, 0.82)	0.44 (0.33, 0.58)	
		**Tertiary**	271	53%	1.15 (0.73, 1.81)	944	42%	0.44 (0.35, 0.56)	0.34 (0.27, 0.44)	
		**Residential or other**	477	42%	0.52 (0.31, 0.88)	1683	45%	0.47 (0.37, 0.60)	0.40 (0.31, 0.53)	
	**Road width**	**Change per 1 m increase**	-	-	1.04 (0.95, 1.12)	-	-	1.11 (1.08, 1.15) ***	1.07 (1.04, 1.11) ***	*p =* 0.19
	**Gradient**	**Change per 1% increase in incline**	-	-	0.97 (0.91, 1.03)	-	-	0.95 (0.92, 0.98) **	0.95 (0.91, 0.98) **	*p =* 0.75
	**Speed limit**	**20 mph or less**	218	48%	1	1026	47%	1	1	*p =* 0.83
		**30 mph**	885	50%	0.83 (0.47, 1.49)	3748	51%	0.97 (0.77, 1.23)	0.96 (0.73, 1.27)	
		**40 mph**	97	54%	0.87 (0.38, 2.02)	298	52%	1.10 (0.74, 1.65)	1.04 (0.63, 1.69)	
		**over 40 mph**	147	52%	1.04 (0.45, 2.43)	200	48%	1.05 (0.66, 1.66)	1.14 (0.64, 2.02)	
	**Connectivity**	**0–24%**	65	40%	1	245	42%	1 *	1	*p =* 0.16
	**rank**	**25–49%**	126	42%	1.03 (0.52, 2.03)	496	43%	1.08 (0.77, 1.53)	1.07 (0.75, 1.52)	
		**50–74%**	260	46%	1.26 (0.66, 2.42)	986	48%	1.36 (0.98, 1.88)	1.28 (0.92, 1.78)	
		**75–100%**	801	54%	1.65 (0.82, 3.32)	3416	52%	1.07 (0.76, 1.50)	0.98 (0.69, 1.39)	
**Road ** **type, ** **second road**	**Road class**	**Primary**	-	-		885	53%		1 ***	-
		**Not primary**	-	-		4429	49%		2.04 (1.63, 2.54)	
	**Road width**	**Change per 1 m increase**	-	-		-	-		1.08 (1.05, 1.12) ***	-
	**Speed limit**	**20 mph or less**	-	-		1100	49%		1	-
		**30 mph**	-	-		3193	50%		1.00 (0.77, 1.29)	
		**40 mph**	-	-		192	53%		0.92 (0.55, 1.56)	
		**over 40 mph**	-	-		158	48%		0.75 (0.42, 1.33)	
**Nearby ** **street ** **infrastructure**	**Bicycle**	**None**	1144	50%	1	4059	48%	1 ***	1 ***	*p =* 0.09
	**infrastructure**	**Track (no lane)**	103	44%	0.81 (0.49, 1.35)	468	55%	1.31 (1.04, 1.65)	1.31 (1.03, 1.67)	
		**Lane (no track)**	74	62%	1.68 (0.92, 3.05)	552	59%	1.52 (1.20, 1.92)	1.60 (1.25, 2.05)	
		**Track and Lane**	9	78%	11.84 (0.88, 159.8)	75	68%	2.23 (1.28, 3.90)	2.34 (1.31, 4.18)	
		**Other, e.g., sign**	13	31%	0.47 (0.12, 1.87)	129	52%	1.50 (1.00, 2.24)	1.36 (0.90, 2.05)	
	**Guardrail**	**No**	1201	49%	1	4397	48%	1	1	*p =* 0.80
		**Yes**	142	59%	1.31 (0.85, 2.00)	886	58%	1.18 (0.99, 1.41)	1.14 (0.94, 1.37)	
	**Bus lane**	**No**	1288	49%	1	4979	49%	1 ***	1 ***	*p =* 0.47
		**Yes**	55	64%	1.84 (0.88, 3.84)	304	68%	1.87 (1.37, 2.54)	1.89 (1.38, 2.58)	
	**Bus stop**	**No**	1212	51%	1 **	4804	50%	1	1	*p =* 0.24
		**Yes**	156	44%	0.57 (0.39, 0.84)	510	49%	0.82 (0.66, 1.00)	0.90 (0.72, 1.12)	
	**Metro/rail/**	**No**	1361	50%	[omitted]	5281	50%	1	1	*p =* 0.99†
	**tram stop**	**Yes**	7	100%		33	64%	1.20 (0.52, 2.76)	1.67 (0.70, 3.98)	
	**Petrol station or**	**No**	1314	49%	1	4945	49%	1 *	1 *	*p =* 0.54
	**car park**	**Yes**	54	63%	1.73 (0.92, 3.22)	369	57%	1.38 (1.08, 1.77)	1.34 (1.03, 1.74)	
	**Traffic signal**	**No**	-	-		4833	49%		1	-
		**Yes, no ASL**	-	-		303	61%		1.14 (0.84, 1.55)	
		**Yes, with ASL**	-	-		178	59%		1.26 (0.87, 1.83)	
	**Roundabout**	**None**	-	-		4559	47%		1 ***	-
		**Roundabout**	-	-		557	69%		2.98 (2.25, 3.95)	
		**Mini-roundabout**	-	-		198	69%		3.55 (2.39, 5.27)	
**Vehicle factors, first road**	**2-way average morning peak speed**	**Change per 10 mph increase**	-	-	0.93 (0.79, 1.10)	-	-	0.76 (0.70, 0.82) ***	0.78 (0.72, 0.85) ***	*p =* 0.004
	**Parked cars**	**No**	584	51%	1	2065	52%	1	1	*p =* 0.87
		**Yes**	759	49%	1.01 (0.76, 1.34)	3218	49%	0.99 (0.86, 1.14)	1.06 (0.91, 1.23)	
	**No. cycle commuters on segment**	**Change per 100 cyclists increase**	-	-	0.95 (0.83, 1.08)	-	-	0.94 (0.90, 0.99) *	0.94 (0.89, 0.99) *	*p =* 0.86
**Vehicle factors, second road**	**2-way average morning peak speed**	**Change per 10 mph increase**	-	-		-	-		1.17 (1.08, 1.27) ***	-

† *p* < 0.1, * *p* < 0.05, ** *p* < 0.01, *** *p* < 0.001 in tests for heterogeneity. ASL = advanced stop lane. Numbers in the N’ column add to less than 1368/5314 points for some variables due to missing data. In all other columns all points are used, using multiple imputation. All models additionally adjust for workplace density, as linear and quadratic terms, and a dummy variable ‘0–5 cycle commuters versus 6+’. † From interaction test in univariable analysis, as multivariable model could not converge. 4 points, from 2 injuries, are excluded because it was not possible to sample a control point matched for intersection status (e.g., as the injury occurred at the first intersection after the participant’s house).

## Data Availability

Many of our datasets are freely available, such as police injury data, and OpenStreetMap data. Other datasets may be available by correspondence with the data owner.

## Supplementary Materials

The following are available online at https://www.mdpi.com/1660-4601/18/6/3060/s1, Table S1: Route environment data sources; Table S2: Results stratified by KSI (killed and seriously injured) status; Table S3: Predictors of injury, all points—with controls selected not matching for intersection status; Table S4: Predictors of injury, among points at intersections (*n* = 5314), according to the combination of the road class of the first road and the second road.
