# Supplementary material for: Association of Infrastructure and Route Environment Factors with Cycling Injury Risk at Intersection and Non-Intersection Locations: A Case-Crossover Study of Britain
Source: Int J Environ Res Public Health. 2021 Mar 16;18(6):3060. doi: 10.3390/ijerph18063060 (PMC8002360; doi:10.3390/ijerph18063060)
Supplement: Supplementary file 1 [file ijerph-18-03060-s001.pdf]

Appendix 1: Route environment data sources

Table A1: route environment data sources

Sequence number	Variables and contributing factors	Value	Type of variable	Operationalisation of variables	Dataset name, owner, and date	Data location
		**A. Area type**				
1	Urban	1 Rural<br>2 Urban	Polygon	We matched the Rural Urban Classification with the boundaries for England, Wales and Scotland using the Lower Layer Super Output Areas code. Then, we identified where the injury and control points are located within the boundaries of LSOA.	Rural Urban Classification, GOV.UK, Department for Environment, Food & Rural Affairs, January 2020<br><br>Urban Rural Classification, Scottish Government, Geographic Information Science & Analysis Team, January 2020	https://data.gov.uk/dataset/b1165cea-2655-4cf7-bf22-dfbd3cdeb242/rural-urban-classification-2011-of-lower-layer-super-output-areas-in-england-and-wales<br><br>https://statistics.gov.scot/data/urban-rural-classification
2	High Street	0 Not on or close to a high street<br><br>1 On or close to a high street	Point	We used the POI catalogue but only some of the categories. These were Retail, Eating and drinking, Education and health, Sport and entertainment, Attractions, Commercial services. Once we selected the classification, we matched them with the corresponding data from the whole POIs dataset and we created polygon the clustering based on the point data using ArcGIS. Then we selected the road network from OSM within the polygon cluster. At the final step, we selected all the injury and control points that are located 25 m near of the selected road network.	Points of Interest, Ordnance Survey, November 2018 dataset used	https://www.ordnancesurvey.co.uk/documents/product-support/support/points-of-interest-classification-scheme.pdf<br><br>https://digimap.edina.ac.uk/
3	Average deprivation	Change per standard deviation increase	Polygon	We located injury and control points inside each zone and looked up the deprivation levels per household for Lower Super Output Areas and Data Zones.	Classification of household deprivation (Great Britain) 2011 - Lower Super Output Areas and Data Zones, UK Data service, dataset used December 2019	https://www.statistics.digitalresources.jisc.ac.uk/
4	Workplace density		Polygon	The workplace population with the boundaries has been matched. Then we located injury and control points inside each zone and looked up the workplace density.	Classification of Workplace Zones, Consumer Data Research Centre, dataset used Janury 2020	https://data.cdrc.ac.uk/

			**B. Road Type**			
5	Road class(hierarchy)	Primary Secondary Tertiary Residential or other	Line	We mapped injury and control points to the nearest OSM road segment. As vector datasets represent roads as lines, and injuries are more frequent on major than minor roads, matching off-network points by distance tends to disproportionately allocate the points to minor roads, at intersection locations (Aldred et al, 2018). We similarly found that when comparing our initial distance-based matching of injury points to route segments, only 31.6% were matched to major roads, compared to an allocation of 43.3% by the police for the same set of points. While police data is not always completely accurate, this disparity suggests that at or close to intersections, our matching was biased towards minor roads. Hence, we carried out the following process. Points lying within 10m of an intersection (267 locations) were reclassified to a major road, where they had initially been assigned to a minor road. In total, this then gave 1472 injury points located at a major road, a number that represents 43.7% of injury points, close to the proportion recorded by the police (43.3%).	Great Britain (England, Scotland and Wales) datasets, Open Street Map, dataset used March 2019	https://www.geofabrik.de/data/download.html
6	Road width	Change per 1m increase	Line	We used the OS Mastermap road network. Then the nearest roads on a range (buffer zone) of 20 m. of the injury and control points were selected. We used the average road width classification from the dataset.	Highways Network Road, Ordnance Survey, November 2019 dataset used	https://www.basemap.co.uk/

7	Gradient	Change per 1% increase in incline	API	The elevations and the distances from the Cyclestreets API have been used. We used road segments up to 250 meters before the injury and control points with the same slope in order to calculate the gradient.	Cyclestreers API, Cyclestreets, journey planner system, APi used March 2020	https://www.cyclestreets.net/api/
8	Speed limit	1 20 mph or less<br>2 30 mph<br>3 40 mph<br>4 over 40 mph	Line	We selected the nearest road on a range (buffer zone) of 20 m. of the injury and control points.	Basemap (the creator of the dataset) directly provided speed limit data from 2017 to us; speed limit data is also now available via Ordnance Survey Public Sector Mapping Agreement: https://www.ordnancesurvey.co.uk/business-government/products/mastermap-highways-speed-data	Basemap (the creator of the dataset) directly provided speed limit data from 2017 to us; speed limit data is also now available via Ordnance Survey Public Sector Mapping Agreement https://www.ordnancesurvey.co.uk/business-government/products/mastermap-highways-speed-data
9	Connectivity rank	0-24%<br>25-49%<br>50-74%<br>75-100%	Line	The SpaceSyntax dataset has been used. It is a linear dataset and we used the 10km Choice Rank classification. The nearest road segment on a range of 20 meters of the injury and control points has been used.	Space Syntax OpenMapping, Spacesyntax, dataset used January 2020	https://spacesyntax.com/openmapping/
		**C. Nearby street infrastructure**				
10	Bike infrastructure	0 No bicycle infrastructure<br>1 Track (no lane)<br>2 Lane (no track)<br>3 Track and Lane<br>4 Other, e.g. sign	GSV	Lookups to see whether any bicycle infrastructure was present at any of the four streetview images that were downloaded for each point (where available). Then coding of the bicycle infrastructure type, separating lanes (on-road, paint-based) from tracks (off-road, separated from motor vehicles in some way).	Google Street View images, Google API, API used November 2019-March 2020	https://rrwen.github.io/google_streetview/<br>https://developers.google.com/maps/documentation/streetview/intro
11	Bus lane	0 Not bus lane<br>1 Yes, bus lane	GSV	GSV lookups to see whether a bus lane was visible in any of the four lookup images.	Google Street View images, Google API, API used November 2019-March 2020	https://rrwen.github.io/google_streetview/<br>https://developers.google.com/maps/documentation/streetview/intro
12	Bus stops	0 No, bus stops in a range of 20m	Point	We used data from NAPTAN. We created the point based on the coordinates and then used a 20 m range	National Public Transport Access Nodes, Department for Transport, dataset used December 2019	https://data.gov.uk/dataset/ff93ffc1-6656-47d8-9155-85ea0b8f2251/national-

		1 Yes, bus stops in a range of 20m		(buffer zone) from injury and control points in order to select all the relative points (Bus stops)		public-transport-access-nodes-naptan
13	Metro/rail/tram stops	0 No, bus stops in a range of 20m<br>1 Yes, bus stops in a range of 20m	Point	We used data from NAPTAN. We created the point based on the coordinates and then used a 20 m range (buffer zone) from injury and control points in order to select all the relative points (Metro/rail/tram stops)	National Public Transport Access Nodes, Department for Transport, dataset used December 2019	https://data.gov.uk/dataset/ff93ffc1-6656-47d8-9155-85ea0b8f2251/national-public-transport-access-nodes-naptan
14	Petrol station or car park	0 Without Petrol station or car park on a range of 20 m<br>1 Within Petrol station or car park on a range of 20 m	Point, polygon	Data from OSM was used. Then we selected all the points that are related to the petrol station or car park in a range (buffer zone) of 20 m from injury and control points.	Great Britain (England, Scotland and Wales) datasets, Open Street Map, dataset used January 2020	https://www.geofabrik.de/data/download.html
15	Intersection	0 Without an intersection in 20 m range<br>1 Within an intersection in 20 m range	Line, point	The OSM road network was used. We identify the intersections using ArcGIS. Then we used a range (buffer zone) of 20 m of the injury and control points that lay near to an intersection.	Great Britain (England, Scotland and Wales) datasets, Open Street Map, dataset used March 2019	https://www.geofabrik.de/data/download.html
			**D. Travel behaviour**			
18	2-way average morning peak speed	Change per 10mph increase	Line	We used the average speed based on 2017 from basemap. We matched the speed data with the Master map network based on the TOID number. Then the nearest road on a range (buffer zone) of 20 m. of the injury and control points were selected	TOIDs (based on 2017) which have the average speed for the morning peak, Basemap (the creator of the dataset) directly provided the average speed data for the morning peak from 2017 to us.	https://www.basemap.co.uk/
19	Parked cars	0 Not on or close to cars parked<br>1 On or close to cars parked	GSV	GSV lookups to see whether parked cars were visible in any of the four lookup images.		https://www.geofabrik.de/data/download.html<br><br>https://rrwen.github.io/google_streetview/<br>https://developers.google.com/maps/documentation/streetview/intro

20	Cycle commuters on segment	Change per 100 cyclists increase	Line	We used the PCT tool which uses Census origin-destination data to allocate commuter cyclists across the route network within England and Wales. The nearest road segment on a range (buffer zone) of 20 meters of the injury and control points has been used. As the PCT does not cover Scotland, we used the stplanr package in R (developed for the PCT) to create cycling volume using data from Census 2011	Cycle commuters, Propensity to Cycle Tool (PCT), dataset used December 2019<br><br>Census Scotland 2011, National Records of Scotland, dataset used January 2020	https://www.pct.bike/<br>https://github.com/ropensci/stplanr<br>https://www.scotlandscensus.gov.uk/

Appendix 2: Additional results

Table A2: Results stratified by KSI (killed and seriously injured) status

Category	Predictor	Level	Adjusted, slight only (N= 5498 points)	Adjusted, KSI only (N= 1184 points)	P for Interaction with KSI status
Area type	Urban	Rural<br>Urban	1<br>1.14 (0.72, 1.81)	1<br>1.26 (0.64, 2.50)	p=0.95
	High Street	No<br>Yes	1*<br>1.28 (1.03, 1.59)	1<br>1.51 (0.90, 2.55)	p=0.66
	Average deprivation	Change per standard deviation increase	0.99 (0.91, 1.08)	1.10 (0.91, 1.33)	p=0.42
Road type	Road class	Primary<br>Secondary<br>Tertiary<br>Residential or other	1***<br>0.67 (0.52, 0.87)<br>0.51 (0.40, 0.63)<br>0.47 (0.36, 0.60)	1<br>0.70 (0.42, 1.18)<br>0.74 (0.45, 1.20)<br>0.67 (0.39, 1.13)	p=0.66
	Road width	Change per 1m increase	1.10 (1.06, 1.13)***	1.12 (1.04, 1.20)**	p=0.39
	Gradient	Change per 1% increase in incline	0.96 (0.93, 0.99)*	0.94 (0.88, 1.00)	p=0.41
	Speed limit	20mph or less<br>30mph<br>40mph<br>over 40mph	1<br>0.86 (0.67, 1.09)<br>1.04 (0.70, 1.55)<br>0.87 (0.55, 1.38)	1<br>1.56 (0.91, 2.69)<br>1.36 (0.61, 3.07)<br>2.21 (0.94, 5.19)	p=0.09
	Connectivity rank	0-24%<br>25-49%<br>50-74%<br>75-100%	1<br>1.05 (0.75, 1.46)<br>1.25 (0.91, 1.71)<br>1.08 (0.77, 1.51)	1<br>1.32 (0.64, 2.73)<br>1.85 (0.92, 3.69)<br>1.87 (0.91, 3.86)	p=0.42
Nearby street infra-structure	Bicycle infrastructure	None<br>Track (no lane)<br>Lane (no track)<br>Track and Lane<br>Other, e.g. sign	1***<br>1.18 (0.94, 1.49)<br>1.62 (1.28, 2.06)<br>2.16 (1.20, 3.88)<br>1.36 (0.88, 2.08)	1<br>1.10 (0.67, 1.81)<br>1.19 (0.69, 2.05)<br>4.90 (1.13, 21.27)<br>1.74 (0.74, 4.09)	p=0.73
	Guardrail	No<br>Yes	1<br>1.13 (0.95, 1.35)	1<br>1.43 (0.94, 2.18)	p=0.21
	Bus lane	No<br>Yes	1***<br>1.74 (1.28, 2.37)	1**<br>2.79 (1.30, 6.00)	p=0.29
	Bus stop	No<br>Yes	1*<br>0.80 (0.66, 0.97)	1*<br>0.63 (0.40, 1.00)	p=0.51
	Metro/rail/ tram stop	No<br>Yes	1<br>1.86 (0.73, 4.73)	1<br>0.80 (0.15, 4.33)	p=0.45
	Petrol station or car park	No<br>Yes	1**<br>1.40 (1.09, 1.80)	1<br>1.69 (0.97, 2.93)	p=0.77
Travel behaviour	2-way average morning peak speed	Change per 10mph increase	0.77 (0.71, 0.83)***	0.83 (0.70, 0.98)*	p=0.11
	Parked cars	No<br>Yes	1<br>1.04 (0.90, 1.19)	1<br>0.82 (0.60, 1.13)	p=0.13
	No. cycle commuters on segment	Change per 100 cyclists increase	0.95 (0.90, 0.99)*	0.97 (0.85, 1.10)	p=0.74

†p<0.1, *p<0.05, **p<0.01, ***p<0.001 in tests for heterogeneity. All models additionally adjust for workplace density, as linear and quadratic terms, and a dummy variable '0-5 cycle commuters versus 6+'.

Table A3: Predictors of injury, all points – with controls selected not matching for intersection status

Category	Predictor	Level	N points	% injury points	Univariable	Adjusted 1	Adjusted 2	Adjusted 3
**Area Type**	**Urban**	**Rural**<br>**Urban**	490<br>6,192	45%<br>50%	1***<br>2.02 (1.43, 2.85)	1***<br>1.88 (1.33, 2.66)	1<br>1.37 (0.90, 2.08)	1<br>1.40 (0.90, 2.17)
	**High Street**	**No**<br>**Yes**	6,014<br>668	48%<br>67%	1***<br>2.52 (2.08, 3.06)	1***<br>2.15 (1.77, 2.62)	1***<br>1.75 (1.40, 2.20)	1**<br>1.48 (1.17, 1.86)
	**Average deprivation**	**Change per standard deviation increase**	-	-	1.09 (1.01, 1.16)*	1.09 (1.01, 1.17)*	1.04 (0.95, 1.13)	1.03 (0.95, 1.13)
**Road type**	**Road class**	**Primary**<br>**Secondary**<br>**Tertiary**<br>**Residential or other**	2,511<br>744<br>1,210<br>2,216	59%<br>49%<br>45%<br>43%	1***<br>0.51 (0.41, 0.63)<br>0.41 (0.34, 0.49)<br>0.40 (0.34, 0.46)		1***<br>0.68 (0.53, 0.87)<br>0.59 (0.47, 0.73)<br>0.64 (0.51, 0.81)	1***<br>0.70 (0.54, 0.90)<br>0.59 (0.47, 0.73)<br>0.50 (0.39, 0.65)
	**Road width**	**Change per 1m increase**	-	-	1.23 (1.20, 1.26)***		1.12 (1.09, 1.16)***	1.11 (1.08, 1.15)***
	**Gradient**	**Change per 1% increase in incline (downhill = negative)**	-	-	0.96 (0.94, 0.99)**		0.97 (0.94, 1.00)*	0.96 (0.93, 1.00)*
	**Speed limit**	**20mph or less**<br>**30mph**<br>**40mph**<br>**over 40mph**	1,257<br>4,582<br>424<br>382	47%<br>51%<br>49%<br>45%	1***<br>1.39 (1.16, 1.67)<br>1.21 (0.92, 1.61)<br>0.94 (0.68, 1.30)		1<br>0.92 (0.73, 1.17)<br>0.87 (0.60, 1.26)<br>0.99 (0.65, 1.50)	1<br>0.93 (0.73, 1.19)<br>1.10 (0.74, 1.62)<br>1.38 (0.88, 2.16)
	**Connectivity rank**	**0-24%**<br>**25-49%**<br>**50-74%**<br>**75-100%**	327<br>620<br>1,281<br>4,170	40%<br>43%<br>46%<br>53%	1***<br>1.14 (0.85, 1.51)<br>1.33 (1.03, 1.72)<br>1.93 (1.51, 2.46)		1<br>1.09 (0.78, 1.52)<br>1.19 (0.88, 1.62)<br>1.03 (0.74, 1.42)	1<br>1.20 (0.85, 1.68)<br>1.43 (1.04, 1.96)<br>1.39 (1.00, 1.94)
**Nearby street infra-structure**	**Bicycle infrastructure**	**None**<br>**Track (no lane)**<br>**Lane (no track)**<br>**Track and Lane**<br>**Other, e.g. sign**	5,209<br>571<br>627<br>66<br>131	48%<br>53%<br>60%<br>88%<br>54%	1***<br>1.32 (1.09, 1.59)<br>1.84 (1.51, 2.23)<br>9.55 (4.34, 21.0)<br>1.34 (0.94, 1.91)		1***<br>1.13 (0.90, 1.41)<br>1.34 (1.06, 1.69)<br>5.99 (2.55, 14.0)<br>1.14 (0.75, 1.73)	1***<br>1.17 (0.93, 1.48)<br>1.39 (1.10, 1.76)<br>6.35 (2.61, 15.4)<br>1.22 (0.80, 1.88)
	**Guardrail**	**No**<br>**Yes**	5,704<br>900	47%<br>66%	1***<br>2.33 (1.99, 2.73)		1***<br>1.57 (1.31, 1.89)	1***<br>1.48 (1.23, 1.79)
	**Bus lane**	**No**<br>**Yes**	6,250<br>354	49%<br>69%	1***<br>2.56 (1.99, 3.29)		1**<br>1.58 (1.17, 2.14)	1*8<br>1.60 (1.18, 2.17)
	**Bus stop**	**No**<br>**Yes**	5,987<br>695	51%<br>45%	1*<br>0.81 (0.69, 0.95)		1*<br>0.82 (0.68, 0.99)	1*<br>0.82 (0.68, 1.00)
	**Metro/rail/ tram stop**	**No**<br>**Yes**	6,640<br>42	50%<br>67%	1*<br>2.00 (1.05, 3.80)		1<br>1.30 (0.60, 2.84)	1<br>1.17 (0.53, 2.57)

	**Petrol station or car park**	**No**<br>**Yes**	6,289<br>393	49%<br>63%	1***<br>1.82 (1.46, 2.27)		1**<br>1.50 (1.16, 1.95)	1**<br>1.47 (1.13, 1.92)
	**Intersection**	**No**<br>**Yes**	2,400<br>4,282	29%<br>62%	1***<br>4.42 (3.90, 5.00)		1***<br>3.59 (3.14, 4.10)	1***<br>3.43 (2.99, 3.93)
**Travel behaviour**	**2-way average morning peak speed**	**Change per 10mph increase**	-	-	0.71 (0.67, 0.75)***			0.76 (0.70, 0.83)***
	**Parked cars**	**No**<br>**Yes**	2,832<br>3,772	48%<br>51%	1*<br>1.15 (1.03, 1.28)			1***<br>1.35 (1.17, 1.55)
	**No. cycle commuters on segment**	**Change per 100 cyclists increase**	-	-	1.00 (0.96, 1.04)			0.95 (0.90, 0.99)*

†p<0.1, *p<0.05, **p<0.01, ***p<0.001 in tests for heterogeneity. Numbers in the N' column add to less than 6682 points for some variables due to missing data. In all other columns all 6682 points are used, using multiple imputation. All adjusted models additionally adjust for workplace density, as linear and quadratic terms, and when examining number of commuters on the segment we additionally included a dummy variable '0-5 cycle commuters versus 6+'

Table A4: Predictors of injury, among points at intersections (N=5314), according to the combination of the road class of the first road and the second road

**Predictor**	**Level**	**N points**	**% injury points**	**Adjusted for area, road type, nearby infrastructure and travel behaviour**
**Road class combination**	**Primary 1st * Primary 2nd**<br>**Primary 1st * Minor 2nd**<br>**Minor 1st * Primary 2nd**<br>**Minor 1st * Minor 2nd**	729<br>1,398<br>156<br>3,031	54%<br>60%<br>48%<br>44%	1***<br>2.41 (1.88, 3.08)<br>0.67 (0.44, 1.02)<br>0.83 (0.63, 1.09)

Adjusted model includes the same variables as 'Adjusted 3' in Table 3 of the main text
